# The Association between Smoking and Ectopic Pregnancy: Why Nicotine Is BAD for Your Fallopian Tube

**DOI:** 10.1371/journal.pone.0089400

**Published:** 2014-02-20

**Authors:** Andrew W. Horne, Jeremy K. Brown, Junko Nio-Kobayashi, Hazirah B. Z. Abidin, Zety E. H. A. Adin, Lyndsey Boswell, Stewart Burgess, Kai-Fai Lee, W. Colin Duncan

**Affiliations:** 1 MRC Centre for Reproductive Health, The Queen’s Medical Research Institute, The University of Edinburgh, Edinburgh, United Kingdom; 2 Laboratory of Histology and Cytology, Hokkaido University Graduate School of Medicine, Sapporo, Japan; 3 Moredun Research Institute, Pentlands Science Park, Penicuik, United Kingdom; 4 Department of Obstetrics and Gynaecology, Li Ka Shing Faculty of Medicine, The University of Hong Kong, Pokfulam, Hong Kong, China; Hokkaido University, Japan

## Abstract

Epidemiological studies have shown that cigarette smoking is a major risk factor for tubal ectopic pregnancy but the reason for this remains unclear. Here, we set out to determine the effect of smoking on Fallopian tube gene expression. An oviductal epithelial cell line (OE-E6/E7) and explants of human Fallopian tubes from non-pregnant women (n = 6) were exposed to physiologically relevant concentrations of cotinine, the principle metabolite of nicotine, and changes in gene expression analyzed using the Illumina Human HT-12 array. Cotinine sensitive genes identified through this process were then localized and quantified in Fallopian tube biopsies from non-pregnant smokers (n = 10) and non-smokers (n = 11) using immunohistochemistry and TaqMan RT-PCR. The principle cotinine induced change in gene expression detected by the array analysis in both explants and the cell line was significant down regulation (P<0.05) of the pro-apoptotic gene BAD. We therefore assessed the effect of smoking on cell turnover in retrospectively collected human samples. Consistent with the array data, smoking was associated with decreased levels of *BAD* transcript (*P*<0.01) and increased levels of *BCL2* transcript (*P*<0.05) in Fallopian tube biopsies. BAD and BCL2 specific immunolabelling was localized to Fallopian tube epithelium. Although no other significant differences in levels of apoptosis or cell cycle associated proteins were observed, smoking was associated with significant changes in the morphology of the Fallopian tube epithelium (*P*<0.05). These results suggest that smoking may alter tubal epithelial cell turnover and is associated with structural, as well as functional, changes that may contribute to the development of ectopic pregnancy.

## Introduction

Ectopic pregnancy occurs in 1–2% of all pregnancies in Europe and the United States [1]. In the Western world, it remains the most common life-threatening early pregnancy complication [1], [2].

Over 98% of ectopic pregnancies implant in the Fallopian tube but the aetiology of tubal implantation is largely unknown [3]. Nevertheless, descriptive observations support the hypothesis that tubal implantation is likely caused by embryo retention within the Fallopian tube due to impaired tubal transport and alterations in the tubal microenvironment allowing early implantation to occur. Transport of the embryo through the Fallopian tube is controlled by a combination of smooth muscle contractility and ciliary beating [4], [5]. The factors that regulate and maintain the normal tubal microenvironment are largely unknown.

Epidemiological studies have shown that cigarette smoking is a major risk factor for tubal ectopic pregnancy (adjusted OR 1.7–3.9) [6]. Animal and human studies have demonstrated effects on oviductal/Fallopian tube function resulting from smoke exposure [7], [8]. Despite these findings, the exact mechanism by which cigarette smoking leads to ectopic pregnancy remains uncertain.

We recently reported that cotinine (an active metabolite of nicotine) increases the expression of prokineticin PROKR1 in the Fallopian tube, a regulator of smooth muscle contractility and a gene thought to be important for intrauterine implantation [9]. We proposed that cigarette smoking attenuates tubal PROKR1 expression resulting in changes in Fallopian tube function, providing a possible explanation for the link between smoking and tubal ectopic pregnancy. In this study, we take this finding forward by investigating how smoking alters global gene expression and function of tubal epithelial cells.

## Materials and Methods

### Human Fallopian Tube and Serum Collection

Ethical approval for this study was obtained from the Lothian Research Ethics Committee (04/S1103/20), and informed written consent was obtained from all of the women participating in the study. Serum samples (10 ml) and Fallopian tube biopsies (2–3 cm) from the ampullary region of the Fallopian tube were collected from participants at the time of hysterectomy for benign gynaecological conditions. Women were between 18 and 45 years of age. All biopsies were collected in the mid-luteal phase of the menstrual cycle, and cycle phase was later confirmed by estradiol and progesterone levels measured in the serum samples as described previously [10]. A smoking history was obtained from all patients. In the first part of the study biopsies from non-smokers (n = 3) were transferred to the laboratory in phosphate-buffered saline (PBS) for explant culture [9]. In the second part of the study biopsies (n = 21) were divided into equivalent portions and i) immersed in RNAlater (Ambion, Texas, USA) at 4°C overnight and then flash frozen at −80°C for RNA extraction, or ii) fixed in 4% neutral-buffered formalin overnight at 4°C followed by storage in 70% ethanol, and subsequent embedding in paraffin wax for immunohistochemical staining. Serum samples were stored at −20°C until analysis. Altman's nomogram indicates that a sample size of 8 in each group will have 80% power at the 5% significance level to show a difference of ≥1.4 standard deviations between the two groups, if one truly exists.

### Measurement of Serum Cotinine Concentrations

Serum cotinine concentrations were measured using the direct cotinine ELISA kit (Immunalysis, Pomona, CA), according to the manufacturer's instructions. ELISA data were analyzed in conjunction with the smoking history provided by the participants. A very strong relationship was observed between serum cotinine concentrations and the self-reported smoking status of the patients [9]. All smokers had serum cotinine concentrations in excess of 160 ng/ml while the concentration in the serum of non-smokers did not exceed 12 ng/ml, confirming that cotinine is a good biomarker for smoking. As non-smokers have serum cotinine levels less than 40 ng/ml [9] we used this cut off to divide the samples into two groups: a) non-smokers (cotinine <40 ng/ml) (n = 11) and b) smokers (cotinine >40 ng/ml) (n = 10).

### Exposure of the Immortalised Oviductal Epithelial Cell Line (OE-E6/E7) to Cotinine

OE-E6/E7 cells [11] were maintained in DMEM/F12 medium supplemented with 10% fetal bovine serum (growth medium) in 5% CO2 at 37°C. Cells were seeded at 500,000 cells per well in 12-well dishes and incubated for 24 hours. The growth medium was then removed and cells washed once with PBS, after which serum-free DMEM/F12 (maintenance medium) was added and the cells maintained overnight. The cells were then exposed to 40 ng/ml cotinine (n = 3) (Sigma-Aldrich, Dorset, UK), which represents cotinine concentrations found in the serum of passive smokers, and 400 ng/ml (n = 3), the average concentration found in the serum of active smokers [12]. All cells, including controls, (n = 3) were treated with an equivalent amount of ethanol (0.16% v/v) to control for the cotinine diluent. Cells were treated for 8 hours (the time taken for changes in gene expression to be observed in our previous study) [9] and medium was then removed and cells harvested into 300 µl of RLT buffer (Qiagen, West Sussex, UK) containing 10 µl/ml β-mercaptoethanol and stored at −80°C until RNA extraction. All experiments were performed in triplicate (and replicates pooled). RNA was extracted using the RNA easy kit (Qiagen, West Sussex, UK), according to the manufacturer's instructions which included a DNase treatment step. RNA concentrations were then quality tested and quantified using a Nanodrop Spectrophotometer (Thermo Scientific, Wilmington, DE).

### Exposure of Human Fallopian Tube Explants to Cotinine

Fallopian tube explant culture was performed as previously described [9]. Explants were exposed to 0 ng/ml (n = 3), 40 ng/ml (n = 3) or 400 ng/ml cotinine (n = 3) and again treated with equivalent amounts of ethanol to control for the cotinine diluent. Treatments were performed on duplicate explants (which were later pooled) for 8 hours, at which time the culture medium was removed and tissues placed in 300 µl of Trizol reagent (Invitrogen, Paisley, UK) and frozen at −80°C until RNA extraction. RNA was extracted using Trizol (Invitrogen) according to the manufacturer's instructions. After RNA extraction, DNAase treatment was performed followed by sample clean-up using the RNAeasy kit (Qiagen, West Sussex, UK). After extraction, RNA concentrations were quantified as described above.

### Microarray Analysis of OE-E6/7 Cells and Fallopian Tube Explants Exposed to Cotinine

The effect of cotinine on cell and tubal gene expression was examined using Illumina Human HT-12 microarrays. Array analysis was performed in GeneSpring GX 12.0 (Agilent Technologies). Raw data were pre-processed to remove variability across and within array samples. To minimize non-biological variability across arrays raw data was log2 transformed and then quantile normalised. Further downstream filtering of the normalised array data was performed to remove invariant transcripts based on quality flags and normalised expression values. Heat maps based on average fold-changes (FC) for each gene in the array were generated to visualize the level of correlation between the individual samples and treatment groups and showed that the arrays demonstrated a high degree of correlation between samples. Protocols of the experimental procedures, methods of analysis and microarray data are available as supplementary information in the European Bioinformatics Institute's MIAME compliant ArrayExpress database (http://www.ebi.ac.uk/arrayexpress website). Analysis of differential gene expression was performed as previously described [13]. Briefly, after explorative assessment, a rigorous statistical analysis was exploited to identify differentially expressed genes. The four treatment groups [Low Cell (40 ng/ml OE-E6/7: n = 3), High Cell (400 ng/ml OE-E6/7: n = 3), Low Tissue (40 ng/ml explant: n = 3), High Tissue (400 ng/ml explant: n = 3)] were compared to the respective diluent (ethanol) only control samples (n = 3 per group) using pairwise T-test comparisons with a *P*-value cut-off of <0.05 and a fold change cut-off of >1.1. The lists of genes identified by this method were compared between each of the four analytical groups and any gene matches recorded.

### Quantitative RT-PCR for Fallopian Tube Gene Expression

Messenger RNA was extracted from the FT samples as described previously [10] and reverse transcribed into cDNA using random hexamers (Applied Biosystems, Foster City, CA, USA). Quantitative real-time PCR (QRT-PCR) was carried out on the ABI PRISM 7900 heat-cycler sequence detection system (Applied Biosystems) using specific previously-validated primers (Table S1 in File S1) (Eurogentec Ltd., Southampton, UK). Gene expression was related to a *G6PDH* internal control (Applied Biosystems). Negative controls included Taq polymerase omission, RT negative (containing template hRNA but no RT enzyme) and RT water (containing RT but no template RNA). All samples were analyzed in duplicate and a relative comparison was made to human corpus luteum cDNA [14]. All statistical analyses were performed using Prism (GraphPad Software, La Jolla, USA). A two-tailed t-test was used if the data were normally distributed with similar standard deviations otherwise a non-parametric Mann–Whitney test was used. Differences were considered significant when *P*<0.05.

### Immunohistochemistry of the Fallopian Tube

Formal-saline fixed paraffin wax embedded (FFPE) sections were mounted on Snow Coat X-tra charged slides (Surgipath Europe, Peterborough, UK), dewaxed in xylene, rehydrated and subjected to antigen retrieval by pressure cooking for 20 minutes in 10 mM sodium citrate (pH 6.0), before blocking endogenous peroxidase with 3% hydrogen peroxidase (Sigma, Dorset, UK). An avidin–biotin block (Vector Laboratories, Peterborough, UK) and protein block (Dako, Ely, UK) were performed prior to overnight incubation with primary antibodies (Table S2 in File S1). Negative controls included incubation with equivalent concentrations of non-specific immunoglobulins and omission of the primary antibody. Sections were then incubated with biotinylated secondary antibody and ABC-Elite (Vector Laboratories). Positive immunolabelling was visualized using 3,3-diaminobenzidine (ImmPACT DAB: Vector Laboratories). Sections were then counterstained counterstained in Mayer's Haematoxylin and mounted with No. 1.5 glass coverslips using Pertex (Cellpath PLC, Hemel Hempstead, UK).

### Analysis of Immunostaining

Histological images were captured using an Olympus Provis BX2 microscope (Olympus America Inc. Center Valley, PA, USA) equipped with a Canon E0S 30D Microcam camera (Canon Inc Headquarters, Tokyo, Japan). B cell leukemia/lymphoma 2 (BCL2) and BCL2-associated agonist of cell death (BAD) epithelial immunolabelling intensity, and the degree of surface blebbing in the sections stained for BAD, were graded on a four-point scale by two observers blinded to tissue identity, with excellent correlation, and averaged. Statistical analysis was conducted using a Mann–Whitney test. For assessment of number of cleaved caspase 3 and Ki-67 positive cells five fields were identified and captured using a stratified random sampling technique. Using Image J (http://rsbweb.nih.gov/ij/) software with thresholding both the number of nuclei and the number of immunostained cells were counted. Statistical analysis was conducted using a Mann Whitney test.

### Scanning Electron Microscopy

Representative portions of the Fallopian tube biopsies fixed in neutral buffered formalin were dehydrated through a series of aqueous solutions of ethanol (50 to 100% v/v). Scanning electron microscopy was performed as previously described [15]. Briefly, the specimens were critical point dried from liquid CO2 and mounted on carbon-coated aluminum stubs, and coated with evaporated carbon using an Edwards 306 vacuum coating unit. The samples were subsequently examined using a Cambridge Stereoscan S360 electron microscope (KE Developments, UK). The accelerating voltage was 15 kV.

## Results

### Microarray Analysis of Cotinine Treatment on Cells and Explants

**Figure 1:**
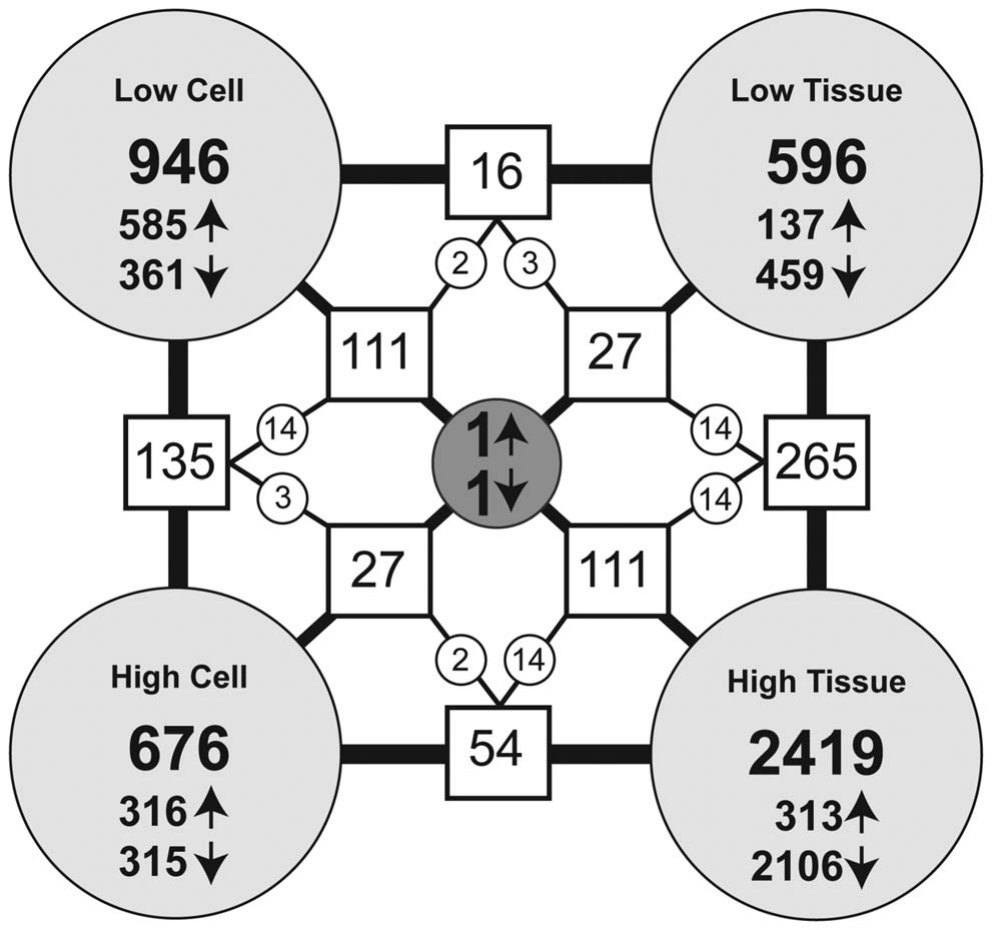
Diagram comparing array data in each of the four analytical groups. In the OE-E6/7 cell line the addition of 40 ng/ml cotinine (‘Low Cell’) altered the expression of 946 genes (increasing 585 genes and reducing the expression of 361 genes). Of the 676 genes (361 increased and 315 reduced) altered by the addition of 400 ng/ml cotinine (‘High Cell’), 135 were shared with the ‘Low Cell’ treatment. In FT explants the addition of 40 ng/ml cotinine (‘Low Tissue’) altered the expression of 596 genes (137 increased and 459 reduced). Explant treatment with 400 ng/ml cotinine (‘High Tissue’) changed 2419 genes (increasing 313 and reducing 2106), 265 of which were shared with the ‘Low Tissue’ treatment. The effect of cotinine in Fallopian tube therefore is mainly to inhibit gene expression. When comparing the effect of cotinine in the OE-E6/7 cells when compared to FT explants common were genes identified. There were 16 altered genes the ‘Low Tissue’ group shared with the ‘Low Cell’ group and 27 genes shared with the ‘High Cell’ group. There were 111 genes that changed expression in common between the ‘High Tissue’ and ‘Low Cell’ group and 54 with the ‘High Cell’ group. There was only one up-regulated gene and one down regulated gene in common to all groups.

Microarray data are available as supplementary information in the European Bioinformatics Institute’s MIAME compliant ArrayExpress database http://www.ebi.ac.uk/arrayexpress website: accession number E-MTAB-12390. After explorative analysis of the microarray data, a rigorous statistical analysis was exploited to identify differentially expressed genes. We then conducted pairwise comparisons between Fallopian tube explant and oviductal epithelial cell lines exposed to each concentration of cotinine and control samples with a p-value cut-off of <0.05 and a fold change cut-off of >1.1, i.e. a 10% difference in expression of up- or down-regulated genes. The lists of genes identified by this method were compared between each of the four analytical groups and any gene matches recorded (summarized in Figure 1). However there was only one up-regulated gene and one down regulated gene in common to all groups. The down-regulated gene highlighted was the pro-apoptotic gene *BAD* and the up-regulated gene was the cell cycle associated gene *NCL*. It therefore seems that both the cell and tissue effects of cotinine *in vitro* relate to regulation of cell turnover. We therefore used this finding as a starting point to assess the effect of smoking on cell turnover in retrospectively collected human Fallopian tube biopsies from smokers and non-smokers, focusing on the BAD/BCL2 pathway.

### BAD Expression is Significantly Reduced in Fallopian Tube of Smokers and BCL2 Expression is Increased When Compared to Non-smokers

**Figure 2:**
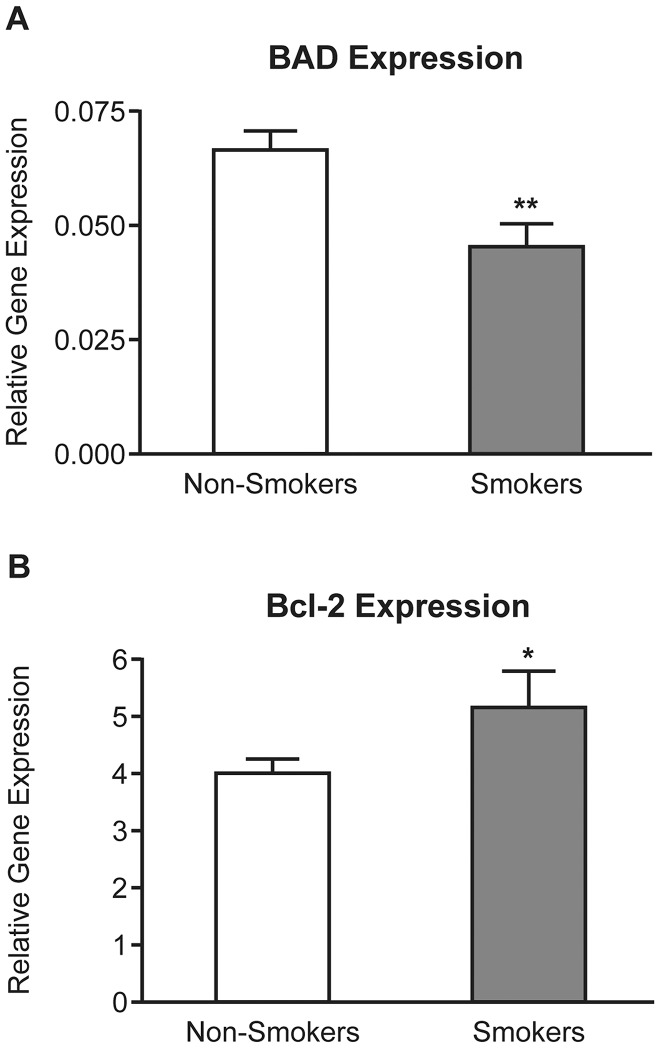
TaqMan RT-PCR analysis of BAD and BCL2 transcript abundance in FT from smokers and non-smokers. Relative expression of *BAD* (A) and *BCL2* (B) in the FT of non-smokers (clear bars: n = 11) and smokers (filled bars: n = 10). Observed differences are significant at * *P*<0.05, **P<0.01. Gene expression was related to a *G6PDH* internal control.

NCL expression was not altered in the FT of smokers when compared to non-smokers (data not shown). However, the expression of *BAD* mRNA was significantly lower (approximately 1.5 fold; *P*<0.01) and expression of BCL2 was found to be significantly higher (approximately 1.5 fold; *P*<0.05) in Fallopian tube from smokers compared to Fallopian tube from nonsmokers (Figure 2). These data suggest an association between FT *BAD* and *BCL2* expression, serum cotinine, and cigarette smoking.

### BAD and BCL2 are Localized to Fallopian Tube Epithelium

**Figure 3:**
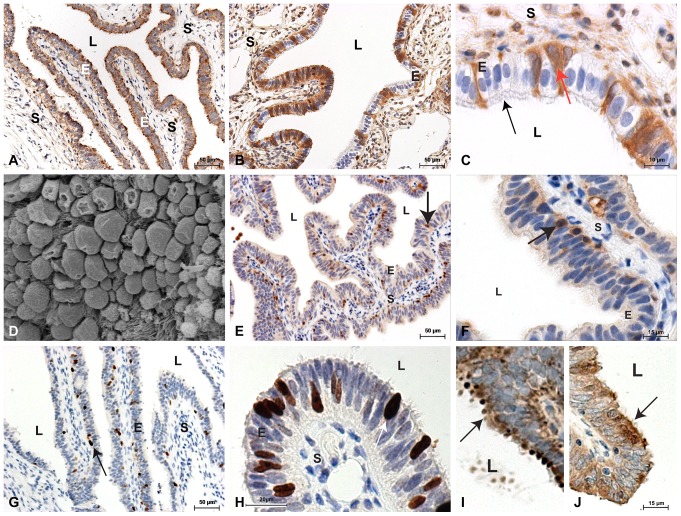
Immunohistochemistry. A) BAD (brown) is expressed in the epithelium (E), most prominently towards the lumen (L), of the FT and not in the stroma (S). B) BCL2 (brown) immunolabelling showing predominant epithelial staining with occasional cell staining in the stroma. C) Higher power BCL2 immunolabelling showing no staining in the ciliated epithelial cells (black arrow) with staining in interspaced cells with no obvious cilia (red arrow). D) Scanning EM highlighting the two populations of epithelial cells with and without cilia. E) Apoptotic cells (brown) identified by immunolabelling for cleaved caspase 3. The arrow shows an apoptotic cell in the tubal epithelium. F) Higher power view showing a cell stained for cleaved caspase 3 (arrow) at the epithelial and stromal junction. G) Cells stained by the proliferation marker Ki-67 (brown). H) Higher power view showing a cell stained for Ki-67 (arrow) in the tubal epithelium. I) Representative image of a section of FT from a smoker immunostained for BAD showing the apical smooth protuberances or ‘epithelial bledding’ (arrow). J) Section of FT immunostained for BAD with the ciliated epithelial cells with no surface ‘epithelial blebbing’.

The epithelium of the ampullary region of the human Fallopian tube was found to express both BAD and the pro-survival gene BCL2 by immunohistochemistry (Figure 3A–C). Although BAD appears to be constitutively expressed in the cytoplasm of all of the epithelial cells, BCL2 is expressed in some epithelial cells and not others. High power analysis shows that it appears to be absent in the ciliated cells (Figure 3B) and expressed in the non-ciliated cells whose surface has a more bleb-like appearance (Figure 3C). These different cell types in the FT epithelium can clearly be seen using scanning EM (Figure 3D).

### Caspases and Cell Death in Fallopian Tube of Smokers Compared to Non-smokers

**Figure 4:**
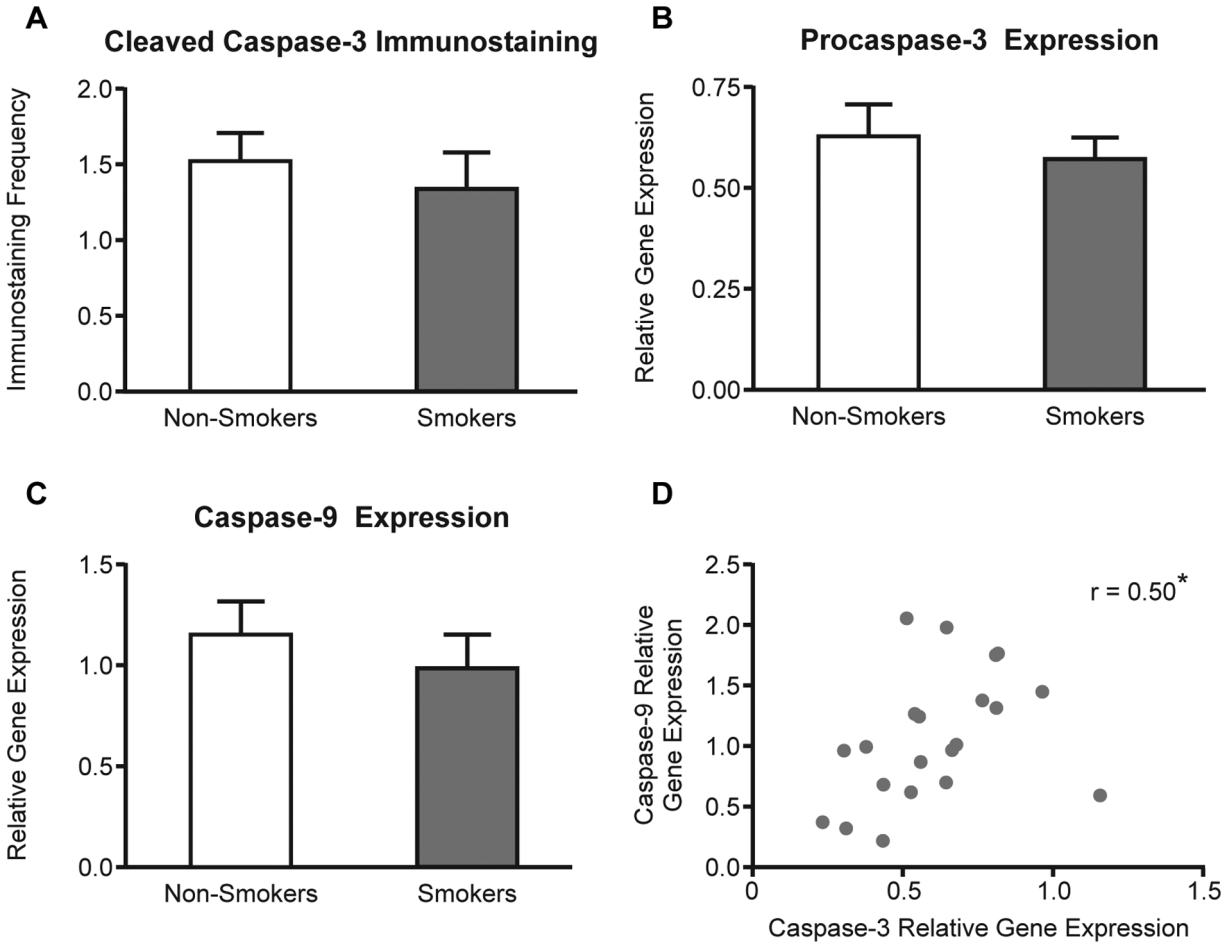
Assessment of cell death in the FT. A) Frequency of immunopositive cells in the FT of non-smokers (clear bars: n = 11) and smokers (filled bars: n = 10). Relative expression of of *CASP3* (B) and *CASP9* (C) transcripts within the FT of smokers and non smokers and (D) their correlation to each other in a single FT sample. Observed differences are significant at * *P*<0.05. Gene expression was related to a *G6PDH* internal control.

In order to investigate the effect of altered *BAD* and *BCL2* expression in the FT we immunolocalized cleaved caspase 3 to identify apoptotic cells in tissue sections (Figure 3E and F). Cells expressing cleaved caspase 3 could be identified in both smokers and non-smokers, and although the numbers were less in smokers this did not reach statistical significance (Figure 4A). Likewise the trend to reduced *CASP3* and *CASP9* expression (Figure 4B and C) did not reach significance although their expression was correlated (r = 0.5, *P*<0.05; Figure 4D). Overall this suggests that there may be a non-significant trend to reduced cell death in the tubal epithelium of smokers.

### Epithelial Cell Proliferation in the Fallopian Tube of Smokers Compared to Non-smokers

**Figure 5:**
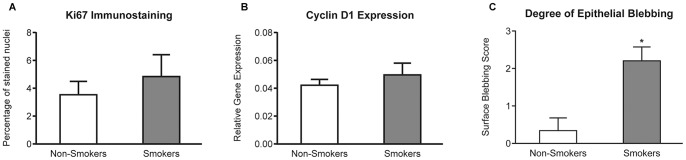
Assessment of changes in cell proliferation and morphology in the FT of smokers. A) Frequency of Ki67 positive cells in the FT of non-smokers (clear bars: n = 11) and smokers (filled bars: n = 10). B) Relative expression of Cyclin D1 transcripts within the FT of smokers and non smokers Expression of *CCND1* in the FT of smokers and non smokers. C) Histoscore analysis of the degree of ‘epithelial blebbing’ in non-smokers compared to smokers. Observed differences are significant at * *P*<0.05. Gene expression was related to a *G6PDH* internal control.

We assessed cell proliferation using immunolocalization of Ki-67 (Figure 3G and H). There were dividing cells in the tubal epithelium from smokers and non-smokers. Although there were more proliferating cells in the FT of smokers this did not reach statistical significance (Figure 5A). In addition the increase in CCND1 (Figure 5B) did not reach significance. Overall however this suggests that there may be a trend to increased cell proliferation in the tubal epithelium of smokers. Taken together with the data on cell death there is a strong suggestion that smoking might affect cell FT epithelial cell turnover. We therefore assessed if this was associated with structural changes of the Fallopian tube.

### Evidence of Cell-surface Irregularity in the Tubal Epithelium of Smokers

The immunolabelling of FT sections with BAD highlighted epithelial features less visible in the negative control sections. Some epithelial cells, and areas of epithelium, had more obvious apical BAD staining in some sections than in others (Figure 3I and J). We termed these epithelial cellular protrusions as ‘epithelial blebbing’ and blind scoring for degree of ‘epithelial blebbing’ on a four point scale indicated that they were significantly higher in Fallopian tube from smokers compared to Fallopian tube from nonsmokers (Figure 5C). This suggests that possible alterations in cell turnover associated with smoking may alter tubal structure and subsequently its function.

## Discussion

Our *in vitro* array studies indicate that cotinine exposure appears to affect expression of genes involved in epithelial cell turnover. We subsequently demonstrate that Fallopian tube biopsies from smokers exhibit evidence of an anti-apoptotic profile (reduced *BAD*, increased *BCL2*), a trend towards increased cellular proliferation and decreased cell death, and structural changes in epithelial cell surface structure. These results suggest that smoking may alter epithelial cell turnover in the Fallopian tube, and the resulting structural alteration may help explain the link between smoking and tubal ectopic pregnancy. Cigarette smoking is also associated with other adverse effects on human reproduction, in addition to tubal ectopic pregnancy, such as infertility and spontaneous abortion, making this is an important finding in the broader context of reproductive health [16].

These results are biologically plausible. Outside the reproductive tract, nicotine in cigarette smoke is well known to pro-survival, inhibiting apoptosis and activating survival pathways in the context of other pathologies, such as lung cancer [17]. In the rat tongue mucosa, *BAD* expression did not change following exposure to cigarette smoke whereas *BCL2* was overexpressed [18]. In addition *BCL2* over-expression in response to cigarette smoke has also been reported in an earlier study in the context of head and neck cancer [19]. The similar findings in oral and respiratory cells and the FT suggest the involvement of a circulating product of smoking rather than a purely topical effect. As this product may be cotinine it would be interesting to study whether nicotine replacement therapy is associated with tubal ectopic pregnancy.

In contrast to our observations in the FT, Hu *et al* [20] reported that cigarette smoke extract has the opposite effect in human airway smooth muscle cells – increasing expression of *BAD* and decreasing expression of *BCL2*. One potential explanation is a topical effect of other constituents of smoke or associated changes in the function of the p53 gene [21] that transcriptionally up-regulates BAD expression by binding to its p53-responsive element [22]. In addition the apoptotic activity and pro-survival activity of BAD and BCL2 are respectively largely determined by their phosphorylation status [23] and nicotine-induced survival may occur through multisite phosphorylation of BAD and phosphorylation of BCL2 [24], [25].

The observed increase in *BCL2* could also be responsible for the decrease in *BAD* transcription. Silencing of *BCL2* leads to the induction of p53-dependent apoptosis in colorectal cancer cells [26]. Therefore, it is possible that cigarette smoking increases *BCL2* expression and that this indirectly leads to a decrease in *BAD* expression through suppression of p53. Whatever the mechanism it is clear that in multiple cell types smoking influences cell turnover. One of the effects of smoking on the FT may be the alteration of the rations of cell death and cell proliferation.

We assessed the effect of the dysregulation of *BAD* and *BCL2* expression by smoking on cell death and cell proliferation in the FT. Overall we did not find clearly significant results. However the trend for cell death, as assessed by cleaved caspase 3 immunolabelling as well as *CASP9* and *CASP3* expression was towards smoking being associated with a reduction. The opposite trend was observed when proliferation was assessed by Ki-67 immunolabelling and *CCDN1* expression. It is possible that the techniques we used are not sensitive enough to pick up clear effects of *BAD* and *BCL2* dysregulation. However, taken together there does seem to be a trend towards altered epithelial cell turnover in the FT as a result of smoking. This is supported by our observation about the structural changes in the epithelium of smokers, an effect highlighted by BAD immunostaining. Although smokers showed more epithelial blebbing than non-smokers, the significance of this is not clear. Blebbing was not apparent in ciliated epithelial cells, which may indicate that smokers have a reduction in cilia and/or ciliated cells. Indeed the up-regulated cell survival gene *BCL2* appeared to be exclusively expressed in the non-ciliated cells of the epithelium and could be accounted for by a relative decrease in ciliated cell numbers. However, a recent retrospective cohort study of the effects of cigarette smoke on epithelial ciliation and ciliogenesis in human FT did not reveal any significant changes in ciliated cell numbers or transcription factors involved in ciliogenesis [27].

Alternatively, changes in the relative levels on BAD and BCL2 may promote an environment suited to embryo attachment in the FT. During intrauterine implantation, the endometrial epithelial and stroma cells avert apoptosis and proliferate, undergoing a process called decidualization, forming an environment that promotes embryo attachment and invasion [28]. A recent study has suggested that the preimplantation uterus relies on the signaling Notch family of transmembrane receptors (specifically Notch1) to inhibit apoptosis and regulate cell cycle progression [28]. It is therefore possible that reduced *BAD* and increased *BCL2* expression in the Fallopian tube, as a result of cigarette smoking, predisposes the tubal microenvironment to implantation through dysregulation of apoptosis and cell proliferation, factors important for embryo receptivity.

We have demonstrated molecular structural effects of smoking on the FT that may affect its function. Notwithstanding the mechanism of these changes, our observations suggest that cigarette smoking appears to promote a pro-survival and anti-apoptotic microenvironment in the Fallopian tube. This coupled with the previously reported effects on prokineticin reception [9] could explain the association between smoking and ectopic pregnancy. We have suggested a possible mechanism by which smoking both alters tubal motility and promotes a tubal environment that is favorable to ectopic implantation.

## Supporting Information

File S1 Table S1. qPCR Primer sequences; Table S2. Primary antibody dilution and detection details.
(DOCX)
