# Supplementary material for: The Association between Smoking and Ectopic Pregnancy: Why Nicotine Is BAD for Your Fallopian Tube
Source: PLoS One. 2014 Feb 20;9(2):e89400. doi: 10.1371/journal.pone.0089400 (PMC3930728; doi:10.1371/journal.pone.0089400)
Supplement: File S1 — Table S1. qPCR Primer sequences; Table S2. Primary antibody dilution and detection details. (DOCX) [file pone.0089400.s001.docx]

**Table S1**

| **Gene** | **Fwd primer 5’-3’** | **Rev primer 5’-3’** |
| --- | --- | --- |
| *BAD* | CTC CTG TGG GAC GCC AGT | GGT AGG AGC TGT GGC GAC T |
| *BCL2* | GCC CTG TGG ATG ACT GAG TA | TTC AGA GAC AGC CAG GAG AAA |
| *CCND1* | AGC TCC TGT GCT GCG AAG TGG AAA C | AGT GTT CAA TGA AAT CGT GCG GGG T |
| *CASP3* | TAG CGG ATG GGT GCT ATT GT | AGG GCT CGC TAA CTC CTC AC |
| *CASP9* | GGA AGA GCT GCA GGT GGA | CTG GCC TGT GTC CTC TAA GC |
| *G6PDH* | CGG AAA CGG TCG TAC ACT TC | CCG ACT GAT GGA AGG CAT C |
| *NCL* | AGG AGG AGG AAG AAG AGG AG | ACA AAG AGA TTG AAA GCC GTA G |

**Table S2**

| **Protein** | **Source** | **Dilution** | **Secondary** |
| --- | --- | --- | --- |
| BAD | Millipore | 1:2000 | Goat anti-rabbit biotinylated (Vector) |
| BCL2 | R&D Biosystems | 1:100 | Rabbit anti-mouse biotinylated (Vector) |
| Cleaved caspase 3 | Cell signaling technology | 1:1500 | Goat anti-rabbit biotinylated (Vector) |
| Ki-67 | Novacastra | 1:200 | Goat anti-mouse biotinylated (Vector) |
